# Forkhead box O1 in metabolic dysfunction-associated fatty liver disease: molecular mechanisms and drug research

**DOI:** 10.3389/fnut.2024.1426780

**Published:** 2024-07-02

**Authors:** Xiangjun Sha, Xinlei Zou, Sidi Liu, Canghai Guan, Wujiang Shi, Jianjun Gao, Xiangyu Zhong, Xingming Jiang

**Affiliations:** General Surgery Department, The 2nd Affiliated Hospital of Harbin Medical University, Harbin, China

**Keywords:** metabolic dysfunction-associated fatty liver disease, transcription factors, forkhead box O1, regulatory role, therapy

## Abstract

Metabolic dysfunction-associated fatty liver disease (MAFLD) is a chronic liver disease that progresses from hepatic steatosis to non-alcoholic steatohepatitis, cirrhosis, and liver cancer, posing a huge burden on human health. Existing research has confirmed that forkhead box O1 (FOXO1), as a member of the FOXO transcription factor family, is upregulated in MAFLD. Its activity is closely related to nuclear-cytoplasmic shuttling and various post-translational modifications including phosphorylation, acetylation, and methylation. FOXO1 mediates the progression of MAFLD by regulating glucose metabolism, lipid metabolism, insulin resistance, oxidative stress, hepatic fibrosis, hepatocyte autophagy, apoptosis, and immune inflammation. This article elaborates on the regulatory role of FOXO1 in MAFLD, providing a summary and new insights for the current status of drug research and targeted therapies for MAFLD.

## 1 Introduction

Metabolic dysfunction-associated fatty liver disease (MAFLD) is a metabolic stress-induced liver injury closely related to insulin resistance and genetic susceptibility. Its pathological changes are similar to alcoholic liver disease and are characterized by liver steatosis, inflammation, hepatic cell damage and varying degrees of fibrosis (1, 2). The disease spectrum includes nonalcoholic fatty liver, non-alcoholic steatohepatitis (NASH) and their related cirrhosis and hepatocellular carcinoma (3). Epidemiological studies show that the current prevalence of MAFLD has reached 25%, this increase in the disease incidence will cause a huge economic burden and be accompanied by an increase in the number of patients with end-stage liver disease and hepatic cancer who require liver transplantation (4). There are currently no guidelines determining the optimal treatment for MAFLD, however lifestyle changes combined with a multi-targeted treatment approaches that target specific triggers (type 2 diabetes, dyslipidemia, obesity, insulin resistance, etc.) may be more effective than single treatment approach. Therefore, there is an urgent need to find MAFLD-related therapeutic targets. Based on comprehensive bioinformatics analysis, FOXO1 is identified as a characteristic target gene in MAFLD (5). Recent studies have shown that various drugs targeting FOXO1 directly or indirectly have been developed and applied in MAFLD. In this review, we focus on the regulatory role of FOXO1 in the occurrence and development of MAFLD, and provide new insights into the diagnosis and treatment of MAFLD.

## 2 Transcription factor FOXO1

The forkhead box (FOX) is a family of transcription factors and 41 genes have been identified in humans so far. This family shares a highly conserved DNA-binding domain (forkhead box domain) consisting of about 100 amino acid residues, which has been proven to be involved in the regulation of cell growth, differentiation, metabolism, and embryonic development (6). Forkhead box O (FOXO) is the O subgroup among the 19 subgroups in the FOX protein family, including four members: FOXO1, FOXO3, FOXO4 and FOXO6. FOXO can specifically recognize two different DNA response elements, DAF-16 binding element (DBE) and insulin-responsive element (IRE) through the forkhead box domain, and the core sequence is 5′-(A/C)AA(C/T)A-3′. Different subtypes of the FOXO family overlap in some functions, but each subtype also has specific physiological and pathological roles. FOXO1 is mainly involved in glucose metabolism, insulin signaling, and lipid synthesis, while FOXO3 is more associated with anti-stress responses, longevity, and cancer. FOXO4 is related to apoptosis and aging, and FOXO6 is primarily expressed in the brain and plays a role in neurodevelopment and function (7). FOXO1 and other FOXO isoforms work synergistically, with their expression and activation being interdependent. Research has shown that FOXO1 induces the expression of RICTOR (rapamycin-insensitive companion of mammalian target of rapamycin), a component of the mTORC2 (mechanistic target of rapamycin kinase complex 2) complex, which mediates the AKT-dependent inactivation of FOXO3 (8). Additionally, FOXO3 can increase the transcription levels of PIK3CA and FOXO1 (9).

**Figure 1:**
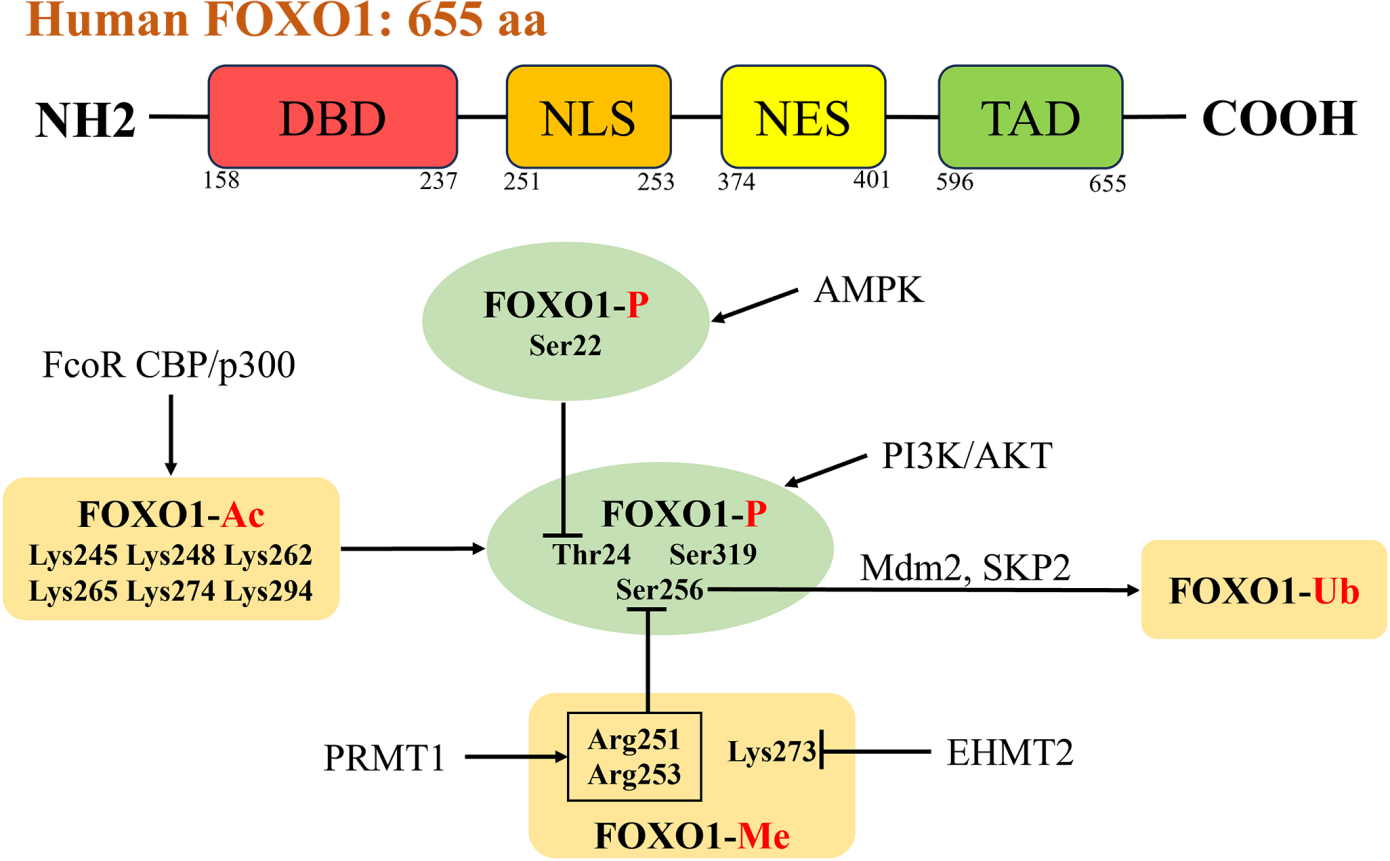
Structure and activity regulation of FOXO1. The human transcription factor FOXO1 consists of 655 amino acid residues, including four functional domains: the NH2-terminal DBD (residues 158–237), the NES (residues 374–401), the NLS (residues 251–253), and the TAD (residues 596–655). There are interactions between different post-translational modifications, and PI3K/AKT signaling pathway is the most typical phosphorylation pathway for FOXO1 (phosphorylation sites Thr24, Ser256, Ser319). Acetylation of FOXO1 enhances AKT-mediated phosphorylation. Methylation of Arg251 and Arg253 inhibits phosphorylation of Ser256. Ubiquitin ligases SKP2 and Mdm2 participate in the ubiquitination and degradation of FOXO1 by binding to phosphorylated Ser256. The phosphorylation of Ser22 mediated by AMPK can interfere with the phosphorylation of Thr24 mediated by AKT. FOXO1, forkhead box O 1; DBD, DNA-binding domain; NES, nuclear export sequence; NLS, nuclear localization signal; TAD, transactivation domain; PI3K, phosphatidylinositol 3-kinase; AKT, protein Kinase B; SKP2, S-phase kinase-associated protein 2; Mdm2, murine double minute 2; AMPK, AMP-activated protein kinase.

The FOXO1 gene was first discovered in human cancer chromosomal translocation studies, located on 13q14.11, and consisted of 3 exons with a total length of 144,267 nt. FOXO1 contains four functional domains, the nh2-terminal DNA-binding domain (DBD, residues 158–237), the nuclear export sequence (NES, residues 374–401), the nuclear localization signal (NLS, residues 251–253), and the transactivation domain (TAD, residues 596–655) (Figure 1), which control nucleoplasmic translocation and gene transcription of FOXO1 (10, 11). The structure of FOXO1 is highly conserved across different species, particularly its DBD and NLS. These conserved domains enable FOXO1 to perform similar functions in various organisms. FOXO1 is closely associated with metabolic regulation, particularly under the control of the insulin signaling pathway. This mechanism has been validated in various organisms, ranging from nematodes to mammals (7). It has been shown that a variety of post-translational modifications including methylation, GlcNAcylation, ubiquitination, phosphorylation and acetylation can activate or inhibit FOXO1 expression, which binds cis-responsive elements of downstream target genes in the nucleus to mediate its transcriptional regulation, whereas its activity is significantly reduced in the cytoplasm, and its cellular distribution is dependent on the internal environment and *in vivo* equilibria (12). FOXO1 facilitates cardiovascular development and prevents pathological remodelling during embryonic development and is the key mediator of exercise-induced physiological cardiac hypertrophy; FOXO1-deficient mouse embryos die around mid-gestation due to abnormal cardiovascular development (13–15). Activation of FOXO1 in neurological diseases improves Huntington’s disease progression and neuronal survival (16). FOXO1 is a well-recognized tumor suppressor gene that can inhibit the proliferation, invasion, and migration abilities of various malignant tumor cells (such as hepatocellular carcinoma, colorectal cancer, gastric cancer, prostate cancer, etc.), while promoting their apoptosis (17). As a cancer suppressor, the loss of FOXO1 activity in tumor-infiltrating regulatory T cells leads to their accumulation in tumor tissues, triggering a strong anti-cancer immune response (18). FOXO1 is expressed in tissues that regulate energy homeostasis, such as the liver, pancreas, skeletal muscle, adipose tissue, and hypothalamus (19). The liver is a key site of action for FOXO1, and compared to normal liver tissue, FOXO1 is highly expressed in MAFLD. It acts as a key regulatory factor mediating physiological and pathological processes such as glucose metabolism, lipid metabolism, insulin resistance, and oxidative stress (17, 20–22).

## 3 Regulation of FOXO1 activity

Post-translational modifications are important pathways for regulating protein function and controlling fundamental physiological processes. Regulation of FOXO1 activity involves a number of modifications including phosphorylation, acetylation, methylation, ubiquitination, glucosylation and glutathionylation (Figure 1). These modifications regulate FOXO1 activity by affecting the subcellular distribution of FOXO1 and DNA-binding affinity (23).

### 3.1 Phosphorylation

Phosphorylation modifications are the most common type of covalent post-translational modifications of FOXO1, and one of the best-known kinase-mediated FOXO1 phosphorylation is phosphoinositide 3-kinase (PI3K)/AKT signaling, in which AKT, a negative regulator, phosphorylates three conserved amino acid residues of the FOXO1 protein, including threonine 24 (Thr24), serine 256 (Ser256), and serine 319 (Ser319), in response to stimulation by insulin or IGF-1 (24, 25). Upon phosphorylation of FOXO1, the 14-3-3 protein binds to the phosphorylation site of FOXO1 to form the FOXO1-14-3-3 complex which in turn affects the subcellular localization of FOXO1, and in the presence of 14-3-3 protein binding FOXO1 normally migrates from the nucleus to the cytoplasm, thus limiting its activity in the nucleus (26–29). Phosphorylated FOXO1 is excluded from the nucleus and this model of FOXO1-mediated nuclear exclusion has been widely recognized (26). Guo et al. have shown that only phosphorylation of Ser256 was necessary for insulin to repress transcriptional activation of FOXO1 (30). However, some of the subsequent experimental data were difficult to explain with the nuclear exclusion model, which was complemented by an intranuclear translocation model proposed by Tamaki Arai’s research, an insulin-dependent translocation model of Ser256 phosphorylated FOXO1 from the nuclear speck of SRSF2 to the perinuclear area (31). In addition to the classical pathway PI3K/AKT, there are also upstream kinases such as AMPK, MST1, CDK1/2, PKA, CK1, CDK4, DYRK1A, NLK, MAPK, and so on. Among them, the phosphorylation of Ser22 mediated by AMPK can interfere with the phosphorylation of Thr24 mediated by AKT, directly and indirectly preventing the binding of FOXO1 to 14-3-3 protein, thereby maintaining the activity of FOXO1 (32). Different kinases target different phosphorylation sites on FOXO1, and the different phosphorylation states correspond to differences in FOXO1 activity (activation/inhibition), thereby affecting biological functions such as glucose metabolism, oxidative stress, cell differentiation, and angiogenesis.

### 3.2 Acetylation

Regulation of FOXO1 activity also involves protein acetylation modifications, and FOXO1 can bind to homology sites on nucleosomes *in vitro*; this binding stably disrupts core histone-dna contacts and results in the opening of chromatin (33). Acetylation of FOXO1 reduces its affinity for binding to DNA, but it does not disrupt the binding of FOXO1 to DNA or has an effect on the stable remodelling of nucleosomes (34, 35). Response elementbinding protein-binding protein and its homologue p300 are histone acetyltransferases involved in the regulation of a variety of pathophysiological processes including oxidative stress, and can acetylate FOXO1 (36, 37). Acetylated FOXO1 can be deacetylated by histone deacetylase (HDAC) with silencing information regulator 1 (Sirt1) and Sirt2, which promotes its nuclear localisation and enhances transcriptional activity (38–43). Furthermore, FOXO1 CoRepressor, a novel FOXO1-binding protein expressed in mouse adipocytes, can directly acetylate FOXO1 *in vitro* by disrupting the interaction between FOXO1 and Sirt1 (44). The two modifications, acetylation and phosphorylation, can be decoupled from each other, with partly overlapping and partly independent regulatory mechanisms for phosphorylation and acetylation (45). Mutations mimicking the acetylation state make FOXO1 more sensitive to AKT-mediated phosphorylation and nuclear rejection and can reverse the constitutive nuclear localisation of phosphorylation-deficient FOXO1, which leads to an increase in the cytoplasmic translocation of FOXO1 and inhibits its activity by indirectly promoting degradation (45, 46). However, the phosphorylation of FOXO1 is not dependent on its acetylation state (45).

### 3.3 Methylation

Protein arginine methyltransferase 1 (PRMT1) methylates multiple proteins and regulates glucose metabolism and stress tolerance. In mice, FOXO1 is methylated by PRMT1 at the Arg248 and Arg250 (equivalent to Arg251 and Arg253 of human FOXO1) sites, and methylation of these residues has an inhibitory effect on AKT-mediated phosphorylation of FOXO1 Ser253 (equivalent to Ser256 of human FOXO1), which promotes FOXO1 entry into the nucleus leading to its enhanced activity. Arginine methylation can act as an antagonistic mechanism to balance FOXO1 phosphorylation (47, 48). In addition, the Lys273 residue of FOXO1 was methylated by euchromatin histone lysine methyltransferase 2 (EHMT2), a histone methyltransferase that regulates apoptotic processes and cell differentiation (49, 50). The different effects of methylation of different FOXO1 residues on protein function indicate the complexity of biological regulation.

### 3.4 GlcNAcylation

GlcNAcylation is a post-translational modification targeting serine/threonine residues involved in the regulation of glucose metabolism, oxidative stress and tumourigenesis. FOXO1 GlcNAcylation mediated by O-GlcNAc transferase (OGT) promotes nuclear translocation of FOXO1 and enhances the transcriptional activity of target genes, such as the gluconeogenesis gene glucose-6-phosphatase (G6Pase), in a manner that is not related to FOXO1 protein expression, AKT-mediated phosphorylation or subcellular distribution (51). Reports indicate that the Thr317, Ser318, Ser550, Thr648, and Ser654 residues are the FOXO1 GlcNAcylated sites and that GlcNAcylated Thr317 affects the transcriptional activity of human FOXO1 (52). However, Fardini et al. revealed that this GlcNAcylation effect is not obvious in mouse experiments (53). The GlcNAcylation protein PGC-1α can act as a co-activator of OGT, enhancing the GlcNAcylation of FOXO1 and its subsequent transcriptional activity (54).

### 3.5 Ubiquitination

FOXO1 activity is controlled by the ubiquitination process. FOXO1 is degraded via the 26S ubiquitin-proteasome pathway in HepG2 and INS-1 cells, and polyubiquitination-mediated degradation is promoted by insulin through PI3K/AKT induced phosphorylation, which leads to cytoplasmic translocation (55, 56). S-phase kinase-associated protein 2 (SKP2) and murine double minute 2 (Mdm2) are E3 ubiquitin ligases involved in FOXO1 ubiquitination degradation and inhibition of FOXO1 activity by binding to AKT-induced phosphorylated Ser256 of FOXO1 (57, 58). Ubiquitin-specific protease 7 (USP7) directly deubiquitinates monoubiquitinated FOXO1 and has an inhibitory effect on the transcriptional activity of FOXO1, leading to decreased expression of downstream genes glucose-6-phosphatase (G6Pase) and phosphoenolpyruvate carboxykinase (PEPCK). USP7 does not affect FOXO1 protein levels and subcellular localisation but reduces FOXO1 DNA binding affinity (59).

### 3.6 Glutathionylation

Cysteine S-glutathionylation is a post-translational modification closely related to oxidative stress, and FOXO1 glutathionylation mainly enhances the dna-binding capacity and transcriptional activity of FOXO1 in oxidative environment without affecting the phosphorylation status and subcellular localisation of FOXO1 (60, 61). However, which of the specific cysteine residues contribute to the effect and how it contributes remains unknown. This requires further exploration of the regulation of glutathionylation of FOXO1.

## 4 The role of FOXO1 in MAFLD

**Figure 2:**
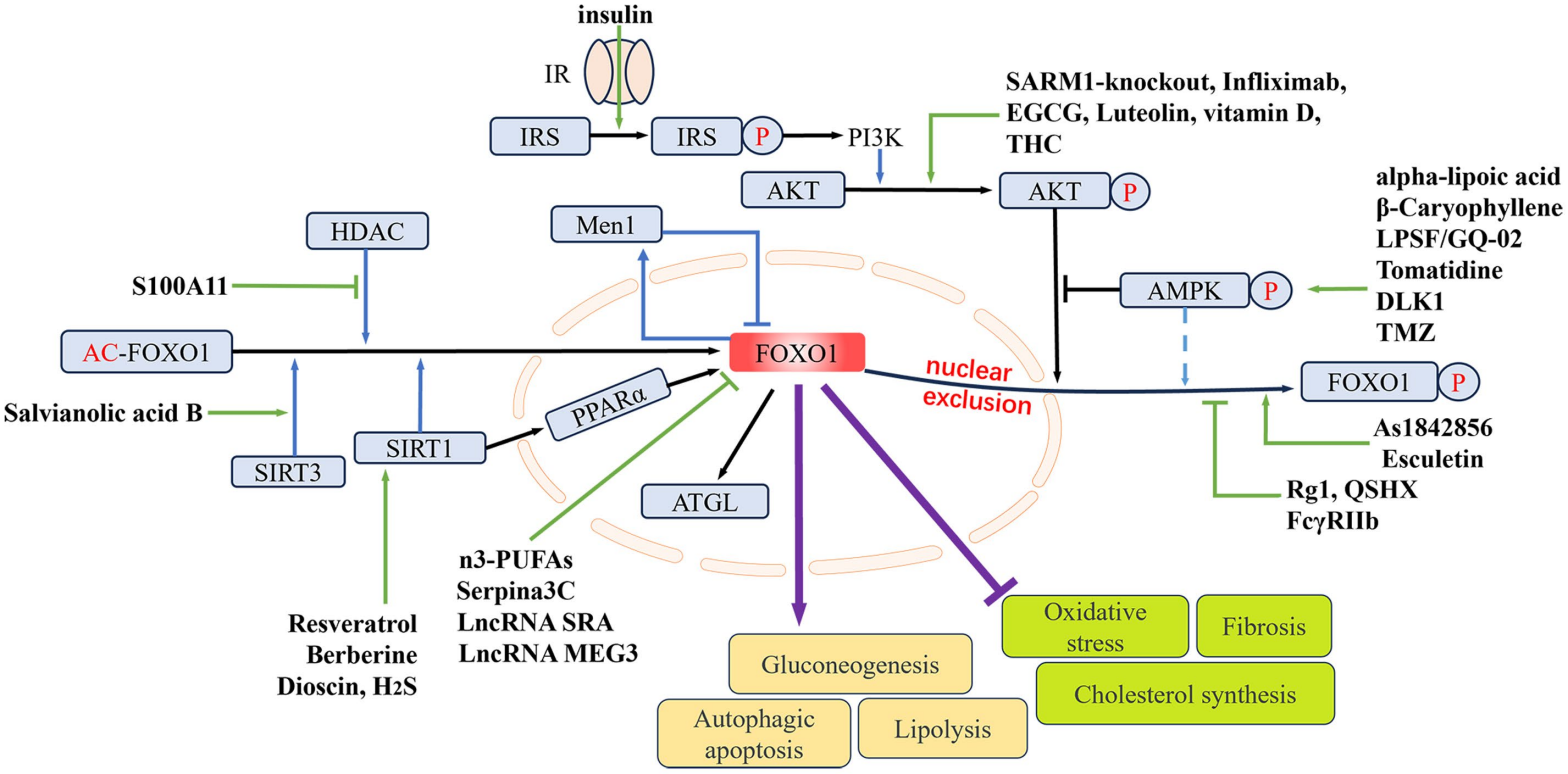
The regulating role of FOXO1 in MAFLD. The nuclear expression of FOXO1 is mainly determined by its phosphorylation and acetylation levels. Phosphorylation of FOXO1 by p-AKT and p-AMPK can lead to its nuclear exclusion. The deacetylases HDAC, SIRT1, and SIRT3 can deacetylate FOXO1, thereby upregulating its nuclear expression. Elevated FOXO1 expression can promote hepatic gluconeogenesis, lipolysis, and hepatocellular autophagy and apoptosis, while inhibiting hepatic oxidative stress, fibrosis, and cholesterol synthesis. Different drugs or genes can directly or indirectly influence nuclear FOXO1 expression, thereby modulating MAFLD. IR, insulin receptor; IRS, insulin receptor substrate; PI3K, phosphoinositide 3-kinases; AMPK, AMP-activated protein kinase; Men1, menin 1; HDAC, histone deacetylase; SIRT1/3, sirtuin 1/3; PPARα, peroxisome proliferator-activated receptor alpha; ATGL, adipose triglyceride lipase; THC, tetrahydrocurcumin; EGCG, epigallocatechin gallate; DLK1, delta like non-canonical Notch ligand 1; TMZ, trimetazidine; LPSF/GQ-02, a thiazolidinone derivative; SARM1, sterile alpha and armadillo motif-containing protein 1; Rg1, ginsenoside Rg1; QSHX, a kind of Chinese medicine extract; H_2_S, hydrogen sulfide; S100A11, S100 calcium binding protein A11; FcγRIIb, Fc-gamma receptor-IIb; n3-PUFAs, n3-polyunsaturated fatty acids; As1842856, a specific inhibitor of FOXO1; LncRNA SRA, long non-coding RNA steroid receptor RNA activator; LncRNA MEG3, long non-coding RNA maternally expressed 3.

FOXO1 plays pivotal roles in MAFLD. As a transcription factor, FOXO1 regulates crucial processes including glucose metabolism, lipid metabolism, inflammation, and fibrosis. During the onset and progression of MAFLD, the function of FOXO1 is regulated by various factors such as nutritional status, insulin signaling pathways, and inflammatory cytokines. Changes in the activity and expression levels of FOXO1 influence the pathophysiological processes in the liver. In clinical settings, increased expression and activity of FOXO1 have been observed in patients with NASH (62, 63) (Figure 2). See Table 1 for details.

**Table 1:** Trial interventions related to FOXO1.

Name	Models	Mechanisms	Therapeutic effects	References
Resveratrol	HepG2 cells	Sirt1 ↑, FOXO1 ↑, SREBP1 ↓	Reduces hepatic lipid accumulation	(64)
β-Caryophyllene	HepG2 cells	AMPK ↑, FOXO1 ↑, p-FOXO1 ↓, ATGL ↑	Promote lipid decomposition and inhibit liver damage	(65)
Alpha-lipoic acid	HepG2 cells	AMPK ↑, FOXO1 ↑, p-FOXO1 ↓, ATGL ↑	Promote lipid decomposition	(66)
LPSF/GQ-02	C57BL/6J mice	AMPK ↑, FOXO1 ↑, p-FOXO1 ↓, ATGL ↑	Promote lipid decomposition and inhibit inflammatory cell infiltration	(67)
Dioscin	AML-12 cells, HepG2 cells, C57BL/6J mice	Sirt1 ↑, p-AMPK ↑, FOXO1 ↑, ATGL ↑	Promote lipid decomposition and inhibit liver damage	(68)
Tomatidine	HepG2 cells	p-AMPK ↑, FOXO1 ↑, ATGL ↑	Promote lipid decomposition	(69)
Sirt1	C57BL/6J mice	PPARα ↑, FOXO1 ↑	Reduces hepatic lipid accumulation	(70)
QSHX	SD rats	p-AKT ↓, p-FOXO1 ↓, FOXO1 ↓	Reduces liver lipid accumulation and damage	(71)
TMZ	HepG2 cells, C57BL/6J mice	p-AMPK ↑, FOXO1 ↓, CHREBP ↓	Inhibit liver lipid synthesis and block the progression of liver fibrosis	(72)
Berberine	HepG2 cells	Sirt1 ↑, FOXO1 nuclear retention, SREBP2 ↓	Inhibits the synthesis of triglycerides and cholesterol	(73)
Tacrolimus	AML12 cells, C57BL/6J mice	FKBP51-FOXO1 complex ↑, HMGCS2 ↓	Inhibit ketosis	(74)
LncRNA SRA	HepG2 cells, ob/ob mice	FOXO1 ↓, ATGL ↓	Inhibits lipid breakdown	(75)
LncRNA MEG3	HepG2 cells	FOXO1 ↓, ACC1 ↓, FAS ↓	Inhibits hepatic lipid synthesis	(76)
DLK1	db/db mice, C57BL/6J mice	p-AMPK ↑, p-AKT ↑, p-FOXO1 ↑, G6pase ↓, PEPCK ↓	Reduce liver lipid accumulation, inhibit gluconeogenesis, and inhibit inflammatory cell infiltration	(77)
FcγRIIb	HepG2 cells, C57BL/6J mice	p-AKT ↓, p-FOXO1 ↓, G6pase ↑, PEPCK ↑	Promotes hepatic lipid accumulation and gluconeogenesis	(78)
Infliximab	Wistar rats	TNF-α ↓, p-AKT ↑, p-FOXO1 ↑, G6pase ↓, PEPCK ↓	Reduces liver inflammation, steatosis, and fibrosis while improving insulin signaling	(79)
THC	HepG2 cells	p-AKT ↑, p-FOXO1 ↑, G6pase ↓, PEPCK ↓	Inhibits hepatic lipid synthesis and gluconeogenesis	(80)
Luteolin	Wistar rats	p-AKT ↑, p-FOXO1 ↑, G6pase ↓, PEPCK ↓， SREBP1 ↓	Reduce serum triglycerides and total cholesterol, inhibit gluconeogenesis	(81)
Vitamin D	C57BL/6J mice	p-AKT ↑, p-FOXO1 ↑, G6pase ↓, PEPCK ↓, SREBP1C ↓, ACC ↓	Inhibits hepatic lipid synthesis and gluconeogenesis	(82)
SARM1-knockout	C57BL/6J mice	p-AKT ↑, p-FOXO1 ↑, G6pase ↓, PEPCK ↓	Inhibit liver damage and insulin resistance, reduce inflammation and oxidative stress	(83)
Men1	NCTC-1469 cells	negative feedback loop between Men1 and fox01	Maintaining stable glucose and lipid metabolism	(84, 85)
Rg1	C57BL/6J mice	FOXO1 ↑, p-FOXO1 ↓	Enhance liver antioxidant capacity and reduce inflammation	(86)
Resveratrol	C57BL/6J mice	FOXO1 ↑, SOD2 ↑	Enhance liver antioxidant	(87)
Salvianolic acid B	SD rats	Sirt3 ↑, Ac-FOXO1 ↓, FOXO1 ↑, SOD2 ↑	Enhance liver antioxidant	(88)
H_2_S	HepG2 cells, C57BL/6J mice	Sirt1 ↑, FOXO1 ↑, SOD2 ↑, PCSK9 ↓	Relieve endoplasmic reticulum stress in liver cells	(89)
EGCG	SD rats	p-PI3K ↑, p-AKT ↑, p-FOXO1 ↓	Improve liver fibrosis	(90, 91)
Esculetin	Wistar rats	p-FOXO1 ↑, TGF-β ↓	Improve liver fibrosis	(92)
S100A11	C57BL/6J mice	Ac-FOXO1 ↑, FOXO1 ↑, ATG7 ↑, LC3-II ↑	Activation of hepatocyte autophagy and lipogenesis	(93)
n3-PUFAs	SD rats	p-IRS-1 ↓, FOXO1 ↓, MAP1LC3B ↓, GAPARAPL1 ↓	Inhibit hepatocyte autophagy	(94)
AS1842856			FOXO1 antagonists	(95)
Serpina3c- knockout	Apoe−/−mice	β-catenin ↓, FOXO1 ↓, TLR4 ↓	Inhibit necroptosis	(96)
exosomal miR-192-5p	SD rats	RICTOR ↓, p-FOXO1 ↓, M1 macrophages ↑	Inducing inflammation	(97)

### 4.1 FOXO1 and lipid metabolism

Disruption in lipid synthesis, transport, and breakdown is one of the primary causes of MAFLD. Various lipid metabolism abnormalities interact in the body, leading to excessive fat accumulation in the liver and ultimately resulting in MAFLD. FOXO1 is a key player in various lipid metabolism pathways in MAFLD. As a transcription factor, it regulates the expression of downstream genes related to lipid breakdown and synthesis, maintaining the balance of lipid synthesis and metabolism in the liver. Ubiquitination-mediated degradation of FOXO1 has been shown to inhibit lipid accumulation in hepatic cells, thus playing a protective role against fatty liver (98). Targeting FOXO1 to regulate lipid metabolism is a current research hotspot in the treatment of MAFLD. Resveratrol is a polyphenolic compound naturally found in plants, it reduces hepatic fat deposition by inhibiting the expression of sterol regulatory element binding transcription factor 1 (SREBP1) through the sirtuin 1 (SIRT1)/FOXO1 signaling cascade (64). As a major active component of clove extract, β-Caryophyllene effectively prevents palmitate-induced lipid accumulation and damage in HepG2 cells. It functions by phosphorylating AMPK at Thr172 through the cannabinoid type 2 (CB2) receptor. This process leads to the translocation of FOXO1 from the cytoplasm to the nucleus. Inside the nucleus, FOXO1 upregulates the expression of ATGL, thereby increasing the β-oxidation of free fatty acids (65). Additionally, Alpha-lipoic acid, the thiazolidinedione derivative LPSF/GQ-02, the natural steroid saponin Dioscin, and the tomato extract tomatine all exhibit similar mechanisms in MAFLD: by inhibiting FOXO1 nuclear exclusion and subsequently upregulating ATGL expression, they promote triglyceride breakdown, thereby reducing fat accumulation within liver cells (66–69). The peroxisome proliferator-activated receptor alpha (PPARα)/FOXO1 signaling pathway is also an important pathway in hepatic triglyceride synthesis. SIRT1 in the liver of MAFLD mice activates the transcription factor FOXO1 by transcriptionally regulating PPARα, thereby inhibiting triglyceride synthesis and impeding the progression of MAFLD (70). High molecular weight adiponectin (HMW APN) is a cytokine that helps delay the progression of MAFLD and is mainly regulated by disulfide-bond A oxidoreductase-like protein (DsbA-L) aggregation. The Chinese herbal extract QSHX inhibits FOXO1 signaling activation to increase the expression of DsbA-L and HMW APN, thereby significantly reducing hepatic steatosis and damage in MAFLD rats (71). Another study has shown that APN improves MAFLD by inhibiting FOXO1 expression through the Akt/FOXO1 signaling pathway. It was found that the Akt1 subtype plays a major role in this signaling pathway (99). Trimetazidine (TMZ) is a drug primarily used to alleviate chest pain associated with angina. In recent years, TMZ has been shown in MAFLD research to reduce hepatic lipid synthesis and block fibrosis progression. After TMZ intervention in palmitic acid-induced HepG2 cells, FOXO1 expression decreases with AMPK activation. The suppressed FOXO1 downregulates the expression and transcriptional activity of carbohydrate-responsive element-binding protein (CHREBP), thereby reducing hepatic lipid synthesis (72). Berberine is a lipophilic alkaloid from the Berberidaceae family, and its protective effect against MAFLD has been confirmed in the recent study. Sterol regulatory element-binding protein 2 (SREBP2) transcriptionally regulates 3-hydroxy-3-methylglutaryl coenzyme A reductase (HMGCR), a classic pathway for cholesterol synthesis. Berberine upregulates the mRNA and protein expression of SIRT1 in palmitic acid-induced HepG2 cells, leading to deacetylation of FOXO1 and promoting its nuclear retention. FOXO1 binds to the IRE sequence in the SREBP2 promoter region in the nucleus, inhibiting SREBP2 transcription and indirectly suppressing HMGCR expression and cholesterol synthesis (73). The muscle-liver-adipose metabolic axis is a recently discovered axis involved in the regulation of hepatic steatosis. Female mice lacking the muscle-specific histone methyltransferase G9a show resistance to obesity and hepatic steatosis induced by a high-fat diet. The potential mechanism involves the muscle-liver-adipose metabolic axis, with the phosphorylation level of FOXO1 playing a key role in this metabolic axis (100). As1842856 is a specific inhibitor of FOXO1, which enhances FOXO1 phosphorylation in the mice liver without significantly affecting total FOXO1 expression. As1842856 has a protective effect against chronic stress-induced hepatic lipid deposition and inflammation. It can inhibit the expression of lipid metabolism-related genes (FAS, FATP and FABP) and cholesterol metabolism-related genes (HMG-CoAR and CYP7A1) (101). Tacrolimus, a macrolide calcineurin inhibitor, is the most commonly used immunosuppressant post-liver transplantation. Li et al. discovered that long-term use of tacrolimus promotes the formation of the FKBP51-FOXO1 complex within hepatocytes, thereby inhibiting the nuclear translocation of FOXO1 and its subsequent binding to the promoter of HMGCS2, the rate-limiting enzyme for ketogenesis. This ultimately results in the low expression of HMGCS2 and reduced ketogenesis. The finding provides a new therapeutic direction for addressing hyperlipidemia and hepatic lipid accumulation induced by long-term tacrolimus use (74).

The studies above indicate that FOXO1, as a transcription factor, regulates lipid metabolism-related genes depending on its subcellular localization and its phosphorylation/acetylation levels. FOXO1 has higher transcriptional activity in the nucleus and lower post-translational modification levels. FOXO1 has multiple effects on MAFLD lipid metabolism; it can upregulate CHREBP to promote hepatic lipid synthesis while also inhibiting SREBP2 to reduce cholesterol production. FOXO1 activation of ATGL to promote hepatic lipid oxidation and decomposition is also a classical pathway for regulating MAFLD lipid metabolism. In addition to the compounds mentioned above, the LncRNA (long non-coding RNA) SRA (steroid receptor RNA activator) can also promote hepatic steatosis by downregulating FOXO1 expression and inhibiting ATGL transcription (75). LncRNA MEG3 (maternally expressed 3) can reduce hepatic lipid accumulation by downregulating FOXO1 expression; however, the underlying mechanisms of this process still require further investigation (76).

### 4.2 FOXO1 and gluconeogenesis/insulin resistance

Insulin resistance is closely related to MAFLD, with up to two-thirds of type 2 diabetes patients having MAFLD. The risk of developing type 2 diabetes is more than doubled in MAFLD patients compared to MAFLD patients, with the highest risk seen in NASH patients. Under normal conditions, insulin promotes hepatic lipid synthesis while inhibiting gluconeogenesis, thereby reducing hepatic glucose production. Insulin resistance selectively inhibits insulin’s glucose-lowering effects. Excessive glucose production leads to elevated blood sugar level, further stimulating insulin secretion and promoting the uptake of free fatty acids and synthesis of triglycerides in the liver, ultimately leading to hepatic steatosis and the development of MAFLD (102). In the formation of insulin resistance, the degree of FOXO1 phosphorylation by upstream AKT decreases, this upregulates the G6Pase expression (key gluconeogenic enzyme) and PEPCK, while downregulating the activity of serum free fatty acid metabolic enzymes, leading to the accumulation of fat in cells (103). Specific knockdown of FOXO1 expression inhibits glycogen breakdown and gluconeogenesis in fasted mice, ultimately leading to hypoglycemia (104). Downregulation of SIRT1 expression in human fetal liver cells leads to decreased phosphorylation levels of AKT and FOXO1. Activated FOXO1 in the nucleus promotes the expression of key gluconeogenic genes, such as G6Pase and PEPCK, inducing an increase in glucose concentration in fetal liver cells (105). Delta like non-canonical Notch ligand 1 (DLK1) is an imprinted gene that regulates mouse adipogenesis and muscle development. After DLK1 triggers AKT phosphorylation, FOXO1 translocates from the nucleus to the cytoplasm, thereby downregulating the expression of G6Pase and PEPCK. This inhibits hepatic glucose production and improves insulin resistance in mice (77). Another example with a similar mechanism is Fc-gamma receptor-IIb (FcγRIIb), which inhibits insulin-induced AKT and FOXO1 phosphorylation, thereby promoting hepatic gluconeogenesis by upregulating G6Pase and PEPCK mRNA expression (78). Tumor necrosis factor alpha (TNF-α) is an inflammatory cytokine that plays a key role in the progression of NASH. Infliximab (anti-TNF-α monoclonal antibody) can reduce hepatic steatosis and fibrosis by upregulating the phosphorylation levels of AKT and FOXO1, thereby improving hepatic insulin resistance (79). Tetrahydrocurcumin (THC), a metabolite of curcumin, exhibits higher activity than curcumin in antioxidant, anti-inflammatory, anticancer, antidiabetic, and neuroprotective effects. *In vitro* experiments have shown that THC can partially reverse the low-level phosphorylation of FOXO1 induced by oleic acid in HepG2 cells by enhance the PI3K/AKT signaling cascade. This suggests that THC may improve glucose consumption imbalance in hepatic cell steatosis and insulin resistance in MAFLD patients. However, this conclusion still needs to be verified by prospective clinical trials (80). Luteolin, when formulated as nanoparticles (NPs) with zinc oxide (ZnO) in Lut/ZnO NPs, exhibits higher bioavailability and solubility. In animal experiments, Lut/ZnO NPs not only reduce the levels of triglycerides and total cholesterol in the serum but also activate the PI3K/AKT/FOXO1 insulin signaling cascade, improving insulin sensitivity in liver cells (81). The association between vitamin D deficiency and many metabolic diseases has been confirmed in previous studies. The beneficial effects of vitamin D are often masked by the pathological consequences of obesity. Intraperitoneal injection of vitamin D in mice can activate the AKT/glycogen synthase kinase 3 (GSK3) and AKT/FOXO1 signaling pathways, increasing hepatic glycogen synthesis and suppressing gluconeogenesis. Additionally, it reduces oxidative stress and inflammation through the nuclear factor kappa B subunit 1 (NFκB) signaling cascade, thereby improving insulin resistance in mice (82). Sterile alpha and armadillo motif-containing protein 1 (SARM1) is a recently discovered cytoplasmic protein containing Toll/interleukin-1 receptor (TIR) domains, involved in toll-like receptor (TLR) signaling. Knockout of SARM1 in mice leads to a significant activation of insulin resistance-related pathways including AKT, GSK3β, IRS1, and FOXO1, thereby alleviating insulin resistance in mice fed a high-fat diet (83). FOXO1 expression in hepatic cells is also regulated by the tumor suppressor gene menin 1 (Men1). Knocking down Men1 leads to increased FOXO1 transcription. There is a negative feedback loop between Men1 and FOXO1, where FOXO1 can bind to the promoter region of the Men1 gene to increase its transcription levels, this helps to maintain cellular homeostasis by coordinating glucose and lipid metabolism between them (84, 85). This indicates that targeting treatments related to the low-level phosphorylation of FOXO1 in insulin resistance can improve insulin sensitivity. Downregulating FOXO1 activity in the insulin signaling pathway could become a new direction for MAFLD treatment.

### 4.3 FOXO1 and oxidative stress

Oxidative stress is considered a major cause of liver damage and disease progression in MAFLD. Liver lipid accumulation affects various sources of reactive oxygen species (ROS), including mitochondria, endoplasmic reticulum, and NADPH oxidase. Oxidative stress reflects an imbalance between the production of ROS and the clearance ability of the antioxidant system. ROS, as byproducts of energy metabolism in different types of hepatic cells, are continuously produced in the liver. Liver lipid overload induces the overproduction of oxidants by affecting ROS generation. High levels of ROS cause oxidative modifications to macromolecules such as DNA, lipids, and proteins, leading to the accumulation of damaged macromolecules and triggering liver damage. This process ultimately drives the progression of MAFLD (106). MAFLD is often associated with progressive aging of liver cells, with the levels of aging-related markers (p21, p53) increasing as the severity of steatosis worsens. The ginsenoside Rg1 component can enhance the liver’s antioxidant potential, inhibit lipid peroxidation, and maintain metabolic homeostasis in a D-galactose-induced mouse aging model, ultimately reducing liver damage and promoting liver anti-aging ability. Quantitative analysis revealed that Rg1 treatment significantly upregulates the expression of FOXO1 in mouse liver cells, while reducing the level of FOXO1 phosphorylation. This suggests that FOXO1 may be involved in enhancing the liver’s anti-aging and antioxidant potential (86). In another study of resveratrol, KKAy mice showed upregulated expression of superoxide dismutase (SOD) and a dose-dependent decrease in the oxidative stress marker malondialdehyde in liver tissue after resveratrol intake. FOXO1 also showed upregulated expression in this context (87). The above experimental results suggest a potential link between FOXO1 and oxidative stress in MAFLD. In a study on NASH, FOXO1 was found to act as a sensing component in the antioxidant signaling pathway. Salvianolic acid B is a polyphenolic antioxidant extracted from *Salvia miltiorrhiza*, it can upregulate the expression of SIRT3 in the liver tissue of NASH rats, thereby reducing the acetylation level of FOXO1. Through the SIRT3/FOXO1 signaling pathway, salvianolic acid B directly activates the mitochondrial endogenous antioxidant enzyme SOD2, promoting the metabolism of reactive oxygen species to counteract oxidative stress, and significantly alleviating the progression of NASH in rats (88). Hydrogen sulfide (H_2_S) is produced in the body through enzymatic or non-enzymatic endogenous reactions and serves as a gasotransmitter in physiological processes. Previous studies have shown that blocking the metabolism of H_2_S could promote hepatic lipid accumulation. Cui et al. further investigated this at the molecular level and found that H_2_S indirectly inhibited proprotein convertase subtilisin/kexin type 9 (PCSK9) transcription by upregulating SIRT1 expression and increasing FOXO1 deacetylation, which helped alleviate lipid-induced endoplasmic reticulum stress in hepatic cells (89).

### 4.4 FOXO1 and liver fibrosis

MAFLD usually progresses dynamically under the stimulation of multiple factors. The progression from NAFL to NASH and liver fibrosis is closely related to a series of liver injuries such as lipotoxicity, oxidative stress, inflammation, and cell apoptosis. These injuries occur sequentially in different liver cells and activate the liver’s regenerative potential, leading to increased deposition of collagen and extracellular matrix, ultimately promoting liver fibrosis. Without intervention, the worsening fibrosis in MAFLD can progress to cirrhosis, liver failure, and hepatocellular carcinoma. Effective intervention to halt the progression of liver fibrosis is essential in preventing MAFLD from developing into end-stage liver disease. Specific knockout of FOXO1 can significantly increase the expression of inflammatory and fibrotic genes in the liver, thereby accelerating liver fibrosis (107). FOXO1 is a key signaling factor that connects cholangiocyte/hepatocyte senescence with MAFLD. *In vitro*, downregulation of cyclin-dependent kinase inhibitor 2A (p16) can reduce the transcription of E2F transcription factor 1 (E2f1) and FOXO1 in cholangiocytes by inhibiting RNA polymerase II and E2f1 binding at the FOXO1 site. This in turn alleviates MAFLD phenotypes such as liver fibrosis, hepatic stellate cell activation, inflammation, and macrophage infiltration in high-fat diet-fed mice (108). Epigallocatechin gallate (EGCG) is a major polyphenol found in green tea, known for its ability to inhibit collagen synthesis and deposition. Studies on extracts from the livers of MAFLD rats indicate that EGCG may reduce FOXO1 phosphorylation levels through the PI3K/AKT signaling pathway, which could be a potential mechanism underlying its anti-fibrotic effects (90, 91). If NASH is not controlled, inflammation and cell damage in the liver will further lead to fibrosis. Supplementing high-fat diet rats with esculetin, a derivative of coumarin, significantly inhibits the expression of transforming growth factor-beta (TGF-β) in the liver tissue, and the De Ritis ratio (AST/ALT) is increased compared to the control group, suggesting that esculetin affects the phosphorylation-related pathways of FOXO1 to improve the fibrotic process induced by high-fat diet MAFLD (92).

### 4.5 FOXO1 regulates cellular autophagy and apoptosis

Autophagy plays a crucial role in the development of MAFLD, as it is believed to promote the formation of lipid droplets and the accumulation of lipids in liver cells. In the tree shrew MAFLD model, the high expression of S100 calcium binding protein A11 (S100A11) inhibits the deacetylation of FOXO1 by directly binding to histone deacetylase 6 (HDAC6), thereby upregulating the acetylation level of FOXO1. This promotes the expression of downstream genes and activates liver cell autophagy and intrahepatic lipid production (93). Omega-3 polyunsaturated fatty acids (n3-PUFAs) can inhibit hepatic steatosis and inflammatory infiltration, and improve insulin resistance in rats by reducing IRS-1 phosphorylation. Additionally, the expression of FOXO1 and its downstream autophagy-related target genes, microtubule-associated protein light chain 3B (MAP1LC3B) and gamma-aminobutyric acid receptor-associated protein-like 1 (GABARAPL1), is reduced. This suggests that n3-PUFAs may inhibit the expression of autophagy-related genes in hepatic cells and thereby hinder the progression of MAFLD by downregulating FOXO1. However, current research on the relationship between n3-PUFAs and autophagy is limited to quantitative measurements, and more direct evidence is needed to validate their association (94). FOXO1 is closely related to apoptosis, after treating primary hepatocytes from normal mice with palmitic acid, the protein expression level of FOXO1 and the degree of necrotic apoptosis in the cells both increase. However, AS1842856 (a FOXO1 antagonist) can significantly downregulate the levels of markers associated with necrotic apoptosis in the cells (95). As a member of the α-1 antitrypsin family, serine proteinase inhibitor, clade A, member 3C (Serpina3c) overexpression is protective against MAFLD progression. Downregulation of Serpina3c expression promotes nuclear localization of FOXO1 and β-catenin under lipotoxic conditions, thereby inhibiting necrotic apoptosis in MAFLD through TLR4 activation (96).

### 4.6 FOXO1 and immunity

In recent years, the association between MAFLD development and immune cells has become a prominent research focus. Liver biopsies from patients with advanced NASH have shown overexpression of FOXO1 in hepatic macrophages. Knockdown of FOXO1 can reduce hepatic macrophage infiltration and induce a shift of macrophages from a pro-inflammatory M1 phenotype to an anti-inflammatory M2 phenotype. This inhibitory effect on macrophages alleviates liver inflammation and insulin resistance induced by a high-fat diet in mice. FOXO1 appears to be a key mediator in activating macrophages and a therapeutic target for preventing MAFLD progression from benign steatosis to NASH (109). FOXO1-low activity CXCR6^+^ CD8^+^ T cells are abundant in the livers of NASH mice and NASH patients. Mechanistically, IL-15 induces FOXO1 downregulation and CXCR6 upregulation, rendering liver-resident CXCR6^+^ CD8^+^ T cells sensitive to metabolic stimuli (including acetate and extracellular ATP) and collectively triggering autoimmunity, exacerbating NASH progression (110). Under conditions of lipotoxic liver injury, hepatocyte-derived exosomal miR-192-5p release increases, activating pro-inflammatory M1 macrophages upon uptake of exosomal miR-192-5p. MiR-192-5p downregulates the protein expression of RICTOR in M1 macrophages, further inhibiting the phosphorylation levels of AKT and FOXO1. This leads to the activation of FOXO1 and induces an inflammatory response by upregulating the activity of pro-inflammatory macrophages (97).

## 5 Conclusion

FOXO1 impacts MAFLD through its involvement in liver glucose and lipid metabolism, insulin resistance, oxidative stress, fibrosis progression, and hepatocyte autophagy and apoptosis. Increasing evidence reveals that the dysregulation of FOXO1 signaling in MAFLD affects downstream target gene expression. Its activity is regulated by various post-translational modifications such as phosphorylation, acetylation, and ubiquitination, as well as nucleocytoplasmic shuttling, which depend on the cell’s metabolic state. FOXO1 is a critical node in many metabolic pathways and physiological processes. Targeted interventions against FOXO1 aim to achieve specific control over abnormal metabolism related to MAFLD, which is of great significance. With further research on FOXO1, more potential mechanisms and diagnostic and therapeutic value in MAFLD are expected to be elucidated, making it a promising biomarker for early diagnosis and potential therapeutic targets of MAFLD.

## Author contributions

XS: Writing – original draft. XlZ: Writing – original draft. SL: Writing – original draft. CG: Writing – review & editing. WS: Writing – review & editing. JG: Writing – review & editing. XyZ: Writing – review & editing. XJ: Writing – review & editing.
